# The complete chloroplast genome of *Allium ferganicum*

**DOI:** 10.1080/23802359.2020.1788454

**Published:** 2020-07-11

**Authors:** Lufeng Liu, Ziyoviddin Yusupov, Hikmatullo Suyunkulov, Zhilin Jiang

**Affiliations:** a College of Agronomy and Biotechnology, Yunnan Agricultural University, Kunming, Yunnan, China; b Institute of Agricultural and Garden Technology, Puer University, Puer, Yunnan, China; c CAS Key Laboratory for Plant Diversity and Biogeography of East Asia, Kunming Institute of Botany, Chinese Academy of Sciences, Kunming, Yunnan, China; d International Joint Lab for Molecular Phylogeny and Biogeography, Institute of Botany, Academy Sciences of Uzbekistan, Tashkent, Uzbekistan; e University of Chinese Academy of Sciences, Beijing, China; f Department Botany and Plant Physiology, Faculty of Biology and Chemistry, Khudjand State University, Khujand, Tajikistan

**Keywords:** *Allium*, chloroplast genome, phylogenetic analysis

## Abstract

The complete chloroplast (cp) genome of *Allium ferganicum* was sequenced and annotated. The whole chloroplast genome consists of 153,126 bp with a typical quadripartite structure separated by a pair of 26,556 bp inverted repeat (IR) regions. The structure also includes the large single copy (LSC) − 81,982 bp and the small single copy (SSC) − 18,033 bp. The *A. ferganicum* chloroplast genome encodes 114 unique genes including 80 protein-coding genes, 30 tRNA genes, and 4 rRNA genes. The phylogenetic trees for 35 plastomes genomes showed that *A. ferganicum* is closely related to *A. sativum* (garlic) and *A. ampeloprasum* (leek).

Species of subgenus *Allium* (Amaryllidaceae) are economically important plants in the genus *Allium* L. in the world, such as leek and garlic, which accounts about 40% (375 species) of the total number species of the genus (Khassanov 2018). *Allium ferganicum* Vved. is an endemic member of subg. *Allium* of Fergana Valley (Eastern Uzbekistan and partly in Tajikistan and Kyrgyzstan). The *A. ferganicum* grows from sea level to about 500 − 800 m altitudes in gypsum-bearing strata. (Khassanov 2017). In this study, *A. ferganicum* cp genome of *A.ferganicum* was successfully assembled and annotated, and its relationship with closely related species (*A. sativum* and *A. ampeloprasum*) were investigated.

The fresh leaves of *A. ferganicum* were collected from Akchop hills, near the Kairakum reservoir, Sultonobod village, Bobojon Gofurov region, Sugd province, Tajikistan (E69^°^92^′^86″, N40^°^31′78″). Voucher specimen has been deposited at Kunming Institute of Botany (ZD0635). Total DNA was extracted from 100 mg of fresh leaves using the modified CTAB method (Doyle and Doyle 1987), then genomic DNA was fragmented by sonication to a size of 350 bp. These libraries constructed above were sequenced by Illumina HiSeq4000 and 150 bp paired-end reads were generated with insert size around 350 bp at Beijing Novogene Bioinformatics Technology Co., Ltd, Beijing, China. The plastid genome was assembled using as a reference *Allium fistulosum* (MH926357, Yusupov et al. 2019) by software NovoPlasty version 3.8.3 (Dierckxsens et al. 2017). The cp genome of *A. ferganicum* was annotated using Geneious v10.2 (Kearse et al. 2012). The annotated chloroplast genome of *A. ferganicum* has been deposited into the GenBank with the Accession number MT588185. RAxML-HPC BlackBox v8.1.24 software (Stamatakis 2006) was used to conduct for maximum likelihood (ML). The phylogenetic trees for 35 plastomes genomes showed that *A. ferganicum* is closely related to *A. sativum* (garlic) and *A. ampeloprasum* (leek) (Figure 1).

**Figure 1.**
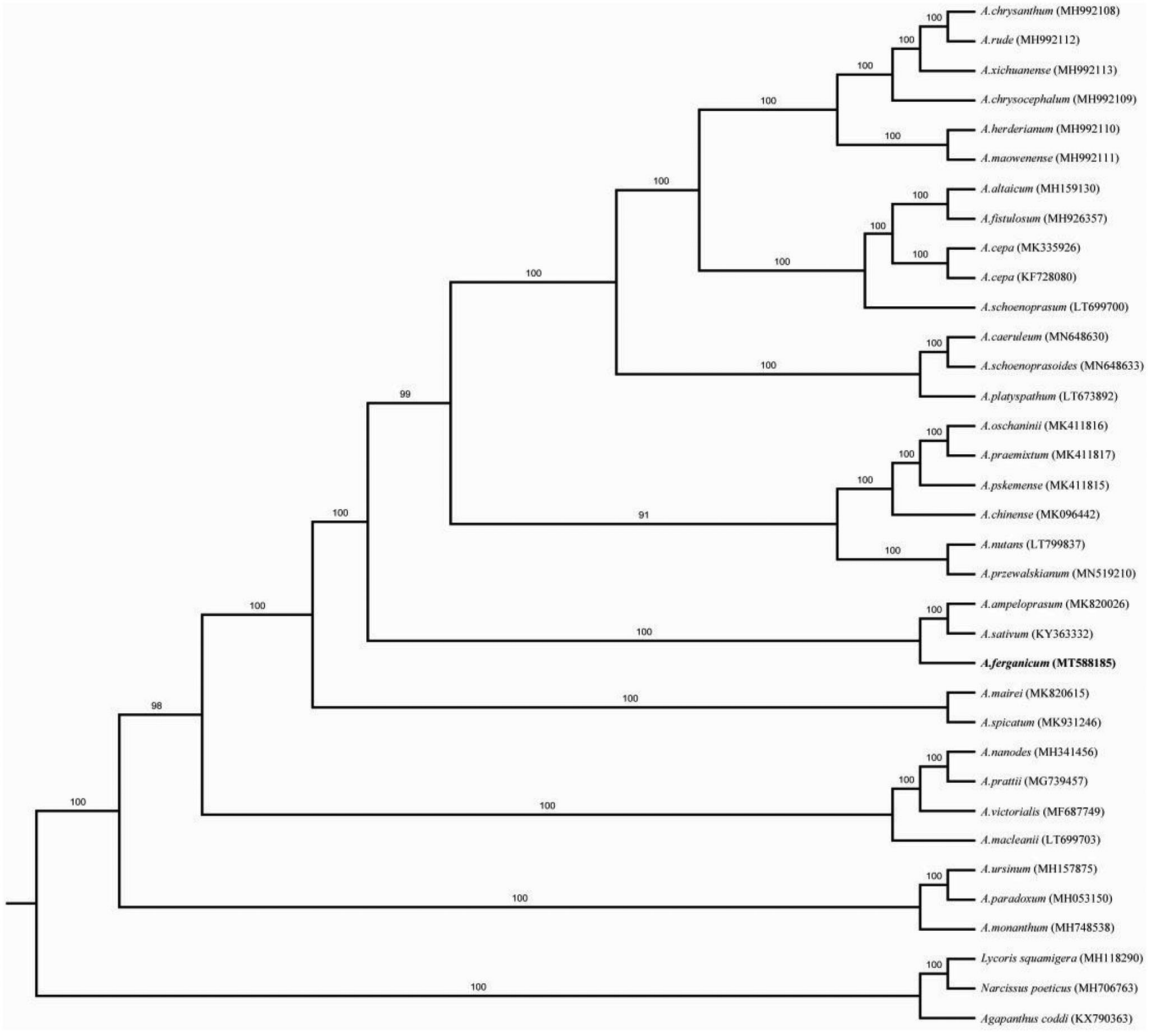
Phylogenetic analysis of *A. fistulosum* with 35 related species. Numbers in the nodes are the bootstrap values from 1000 replicates.

The complete cp genome sequence of *A. ferganicum* is 153,126 bp in length and exhibits a typical quadripartite structure, including a pair of IRs (26,556 bp, GC-42.6% for each) separated by the LSC and SSC regions (LSC: 81,982 bp, GC: 34.5%; SSC: 18,033 bp, GC: 29.1%). The *A. ferganicum* chloroplast genome encodes 114 unique genes including 80 protein-coding genes, 30 tRNA genes, and 4 rRNA genes. Among these, four genes (*rps2*, *rps16* and *ycf15, infA*) were pseudogenes. In the plastome of *A. ampeloprasum* the *infA* gene is absent, unlike the plastome of *A. sativum* and *A. ferganicum*. This plastome sequence of *A. ferganicum* will provide useful plastid genomic resources for population genetics and can be used for phylogenetic analyses of genus *Allium*.

## Data Availability

The data that support the findings of this study are available in [GenBank] at [https://www.ncbi.nlm.nih.gov/genbank], reference number [MT588185].
